# Medical students’ perceptions and understanding of their specific learning difficulties

**DOI:** 10.5116/ijme.524f.cd3f

**Published:** 2013-10-20

**Authors:** Angela Rowlands, Stephen Abbott, Grazia Bevere, Christopher M. Roberts

**Affiliations:** ^1^Barts and the London School of Medicine and Dentistry, Queen Mary University of London, UK; ^2^School of Health Sciences, City University, London, UK; ^3^Dyslexia and Disability Department, Queen Mary University of London, UK

**Keywords:** Specific learning difficulties, academic support, medical students

## Abstract

**Objectives:**

The purpose of this study is to explore how medical students with Specific Learning Difficulties perceive and understand their Specific Learning Difficulty and how it has impacted on their experience of medical training.

**Method:**

A purposive sample of fifteen students from one medical school was interviewed. Framework Analysis was used to identify and organise themes emerging from the data. An interpretation of the data was made capturing the essence of what had been learned. The concept of ‘reframing’ was then used to re-analyse and organise the data.

**Results:**

Students reported having found ways to cope with their Specific Leaning Difficulty in the past, some of which proved inadequate to deal with the pressures of medical school. Diagnosis was a mixed experience: many felt relieved to understand their difficulties better, but some feared discrimination. Practical support was available in university but not in placement. Students focused on the impact of their Specific Learning Difficulty on their ability to pass undergraduate exams. Most did not contemplate difficulties post-qualification.

**Conclusions:**

The rigours of the undergraduate medical course may reveal undisclosed Specific Learning Difficulties. Students need help to cope with such challenges, psychologically and practically in both classroom and clinical practice. University services for students with Specific Learning Difficulties should become familiar with the challenges of clinical placements, and ensure that academic staff has access to information about the needs of these students and how these can be met.

## Introduction

A Specific Learning Difficulty (SpLDs) is an umbrella category that includes dyslexia (reading difficulties), dyspraxia (motor difficulties), dysgraphia (writing difficulties) and dyscalculia (mathematical difficulties). Any of these SpLDs can make study at all levels difficult, although they are not linked with low intelligence.^1^,^2^ Substantial numbers of students who have disclosed a SpLD enroll for higher education^3^ and other students are diagnosed after admission.^4^ Medical schools should expect a significant number of students with SpLDs, as evidence suggests that such individuals are more likely to choose a career in a ‘caring profession’^5^,^6^ indeed, there has been an increase nationally in the number of students diagnosed while at medical school.^7^ Data for the medical school where this study was carried out show that proportionally more medical undergraduate students make themselves known to the university’s Disability and Dyslexia Service than students in other departments.The social model of disability emphasises how individuals with an SpLD are disabled by society’s failure to accommodate their particular needs, thereby further disadvantaging them.^8^ In the UK, national disability legislation ensures that universities address this issue by offering help to students with SpLDs: extra time in examinations, equipment loans and grants for purchasing aids, etc.^9^ Particular issues arise for health care professionals in training because their courses also require extensive experience in clinical practice. Support is likely to vary considerably between these two settings, given their different functions.However, students with SpLDs may do better in the clinical environment: Sanderson-Mann and McCandless^10^ suggest that they tend to have a kinesthetic learning style which makes it easier to learn practical procedures, and, further, that they perform well clinically because of attributes such as creativity, greater oral recall, intuition, multi-dimensional thinking and innovation. Wray et al^11^ and Fink^12^ found high levels of empathy and interpersonal skills among nurses with SpLDs, while Wright’s^13^ study suggested that having a disability can bring with it an insight into what it is like to be ill or disabled, which accordingly promotes the development of caring skills.While the tangible benefits offered in universities give incentives students to disclose their SpLDs, the benefits of disclosure in the clinical areas are less obvious. There is in any case a more general under-reporting of disabilities within the medical profession.^14^ Also; there are difficulties translating strategies learnt in the classroom to the clinical setting. For example, students in one study found that using laptops, which had proved valuable in academic studies, met resistance from clinical staff and caused concern about the safety of the equipment.^15^At an individual level, students have to recognise and accept their SpLD before they can decide whether or not to disclose it and to ask for support. Gerber et al^16^ suggest that in order to achieve, individuals with difficulties need to reframe their SpLD. Re-framing is a process whereby a person changes how they perceive or understand something, leading to changes in their responses and behaviour. Gerber et al^16^ draw on earlier research about people with SpLDs at work to identify four distinct though overlapping stages in the re-framing process: recognition of the SpLD as such, understanding of the nature and implications of the SpLD; acceptance of both negative and positive aspects of the SpLD; and action in pursuit of both short- and long-term goals.A computer based literature search was performed to provide background to the study. A search combined the terms Dyslexia or Dyslex*or Dyspraxia or Dysprax*or Learning difficulties or learning difficult* or learning disabilities or learning disabilit* Nursing students, health professionals (as I wanted to read what work had been done in other health care fields too as certain clinical skill requirements are the same and prevalence is also high in nursing) or medical students.The Library databases used included: PubMed, Web of Science, Cochrane Library, Google Scholar, British Education Index and the British Nursing Index were used (as well as a citation search), as these were considered the most applicable to the area of study. The literature search was limited to evidence in English from the last 10 years. These restrictions were applied since the most relevant legislation has been passed within this time frame. Medical practice is constantly changing and therefore it was important that this study reflected the current climate. In addition, some earlier articles have been included to gain insight into the background and history of the issues.The aim of the present study was to explore how medical students with a SpLD perceived and understood their SpLD and its effect on their education and future careers.

## Method

### Study design

A qualitative methodology (semi-structured interviews) was used for data-gathering, as the emphasis was on self-perceptions rather than objective measurement.

### Participants

Invitations to take part in a semi-structured interview were sent by an e-mail from the Dyslexia and Disability Department to all students attending the medical school who had registered with the university’s SpLD service (N=106). By the three month deadline required by the research timetable, fifteen medical students had volunteered, signed a written consent form, and had been interviewed. The rationale for using this purposive sample^17^ was that it allowed access to and enabled medical students with SpLDs with something to say about their experience to come forward. Guest, Bunce and Johnson^18^ argue that the size of a purposive sample relies typically on the concept of ‘saturation’ of data (the point at which no new information or themes are observed from the data). They found that data saturation occurred within the first 12 interview in qualitative data. Therefore the sample size was deemed large enough to answer the research question.

### Procedure

Interviews were audio-recorded where students agreed (this was included as a separate item on the written consent form.) The students were not personally known to the interviewer prior to the interview. The Queen Mary University of London Research Ethics Committee approved the study. The topic guide included the following topics: becoming aware of SpLD (when, where, feelings, consequences); impact of SpLD on school studies, career choice, university studies (non-medical), studies at medical school, experience on placement; anticipated impact on future career.

### Data analysis

Initially, Framework Analysis was used to identify and organise themes emerging from the data. Ritchie and Spencer^19^ described the 5 key steps of Framework Analysis: familiarisation with the text, identifying a thematic framework, indexing, charting and mapping and interpretation.During the first two stages of the data analysis the interviews were audio-taped and transcribed verbatim. All the data was read so that an overall sense of the information was gained. Each interview was given a number. A thematic framework was then developed. This involved developing an index of the key concepts, issues and themes and organising them into main categories and sub categories. In order to improve rigour inter-rater reliability was used to check the process of analysing the data. Two of us repeatedly listened to the audio records, compared the interviews, identified common themes and transcribed selected sections. We agreed on the meaning of the concepts and themes and compared the similarities and differences in our data analysis. We then developed a single index that we agreed upon and identified new main categories and sub categories.The third stage of the data analysis was indexing (or coding) the data. This was the process of deciding how to conceptually divide up the raw data. Narrative data from the transcriptions was numbered using line numbers so that units of text could be traced back to their original context when needed. Comments from the transcripts were divided up and arranged electronically into groups according to their initial coding before bringing meaning to the information. Certain ideas started to emerge from the transcripts and these were given a preliminary code. Some of these were changed and refined later but they served to begin the process of categorising and analysing the data. Codes were given abbreviations and written next to the appropriate segments of the text as well as recorded in a coding table.In the charting stage the data was synthesised and regrouped. Themes and concepts from all of the transcripts were drawn together. The categories were reduced by grouping topics that related to each other with the aim of generating a small number of themes that would form the main discussion points of the study. The themes were labeled by an expression taken directly from the data. Headings from the thematic framework were used to develop data charts which could be easily read across the whole data set. In the chart boxes, line and page references were put next to the relevant passages in the transcript.In the mapping and interpretation stage an interpretation of the whole data set was made after all the interview transcripts had been coded and charted. The charts and research notes were reviewed at this stage, comparisons and discrepancies between different perceptions and accounts of interviewees were noted and a search was made for patterns and associations within the data. An interpretation of the data was made capturing the essence of what had been learned from the data.It emerged from this process that Gerber et al’s^16^ concept of ‘reframing’ would provide a useful analytic framework, and the data was accordingly re-analysed using Gerber’s stages as themes.

## Results

Fifteen medical students agreed to take part. One refused to be audio-taped but allowed note-taking. Ten were female and five male. Their years of medical training ranged from one to five. Two had previous degrees (one also had a PhD). Ten students described themselves as dyslexic, three as dyspraxic, and one as both; one had been diagnosed as dyslexic and dysgraphic. Interviews took place individually in a private room within the medical school and lasted typically a quarter of an hour (ranging between six and twenty-five minutes).Gerber et al’s^16^ framework was helpful in understanding the data, but it became clear that students with SpLDs were likely to go through the reframing stages more than once at different points in their career, as their learning environment changed. For this reason, we organised the data into three re-framing cycles: before medical school, at medical school, and after medical school.

### Before medical school

Three groups of students could be identified: Group A - those diagnosed with an SpLD before medical school. Group B - those aware before medical school of difficulties associated with learning, but who had not been diagnosed and Group C - those who had not seen themselves before medical school as having learning difficulties.The first group included two (nos. A2 and A4) who had been diagnosed at school, and two (nos. A6 and A7) who were diagnosed during their first degree (though both had been aware of difficulties in learning while at school). The second group (nos. B1, B8, B12, B13, B14, B15) had become aware that they were challenged by some aspects of academic study (e.g. reading, understanding, spelling) more than were their peers, but they had not been diagnosed until medical school. This awareness could be very long-lasting: one said that she had been aware of difficulties when learning her alphabet at primary school, but had not been assessed and diagnosed because of her family’s attitude:

“The sort of background I come from, you don’t really address issues. If you have a learning disability, obviously you just need to work harder; we’ll get you a personal tutor.” *(Interview B12)*

The third group (C3, C5, C9, C10, C11) had not realised that they faced learning difficulties that others did not: they recognised there were weak areas in their academic performance but saw these as normal.Data from group A students suggests that they had experienced the other re-framing stages before medical school; they understood and accepted their SpLDs, and had received help to facilitate study, which enabled them to progress to medical training (the action stage).One student in group A spoke ambivalently about acceptance. Although he did not challenge his diagnosis, and had taken advantage of help, he also believed that he should be trying to overcome his SpLD: he had attempted this before diagnosis, and reproached himself a little for having reduced his efforts since.

“I’m not sure it was a good thing to get diagnosed. I’ve always struggled with spelling and reading and writing, but I’ve always tried to overcome it. But when I got diagnosed, I thought, OK, that explains it, and I sort of stopped trying.” *(Interview A7)*

He thus suggested a dissonance between his behaviour (more accepting) and his belief (less accepting). He did know that expert opinion was that he could not overcome it, but he was reluctant to accept this:

“They say, we’ll teach you coping strategies. But you know, I’ve been coping, I don’t need coping strategies … I want to overcome it, and they say I can’t.” *(Interview A7)*

Given that groups B and C had not recognised their SpLD, one would not expect them to describe the other stages. However, some did speak of what they now realised was their earlier lack of understanding:

“Throughout school, the problems I was facing, I just thought they were the normal problems everyone would face ... I didn’t realise that that amount of time that [I needed] was not normal…English was just a weak subject… I didn’t really take any notice.” *(Interview C11)*

One student in group C itemised two factors that had obscured the problem. Firstly, he had adopted coping strategies without realising that that is what they were:

“I’ve always picked subjects that played to my strengths.” *(Interview C10)*

He understood this as a normal set of preferences for some subjects over others. Secondly, he, his brother and his parents had recently found out that they were all dyslexic, and with hindsight, he realised that this had made his difficulties seem normal.

“You kind of hear all these things and you think that that’s just normal, cos you don’t know any different… so it never occurred to me before…” *(Interview C10)*

Diagnosis at medical school had been enlightening, enabling students to re-frame their past experiences.

“When I got my diagnosis, a lot of things in my past [education and work] came clear… it answered a lot of questions…” *(Interview B8)*

Having a proper assessment could be vital to recognition. One student spoke of how teachers had repeatedly suggested that she was dyslexic, but she had always rejected this idea, as she was consistently good at English and reading: her diagnosis was in fact dyspraxia, and

“suddenly it all made sense.” *(Interview C9)*

### At medical school

By definition, all those interviewed now recognised their SpLD, as they had made themselves known to the SpLD service. For students in groups B and C, it was recognition of the difficulties in studying at medical school that had led to self-referral to the service. Some were disappointed by the grades they were getting; others compared themselves with their peers.

“I was just taking much longer than the other students to write up a lecture, process information … exercises where I would be sharing a handout or a pamphlet or something, I would be like still there minutes after everyone else had finished.”*(Interview B8)*

Some students explained how the increased pressure of medical school had for the first time forced recognition on them.

“I got by [before] by being able to remember stuff and working at my own pace, probably. It was only when I was under so much pressure studying medicine - I was working absolutely flat out, and I think that’s when my strategies that had got me through like just wouldn’t cope any more, and I needed a new way to look at things.’ *(Interview B1)*

Recognition led to understanding of the SpLD and what it took to continue to study.

“I now know that simply writing out things from text books isn’t going to do it for me. And so the module after I had my test result back from the dyslexia person, I did everything in pictures … and then I got top of the class, from being an average person.” *(Interview B8)*

Others spoke of how understanding had an emotional effect.

“I felt really down about it… it dampened my confidence a little bit, so I think my motivation dwindled.” *(Interview 12)*

More mixed feelings were also reported:

“I was actually kind of relieved that it wasn’t something that I was actually doing wrong… [but] I get depressed a lot… I put a lot of effort into work and uni, and the fact that I’m not getting the same return as other people, it’s really frustrating.” *(Interview B13)*

Student B1 had gone for an initial screening by the SpLD service, but had then delayed going for full assessment:

“I had a lot of emotional feelings about having some kind of problems, so I put that off for quite a while.” *(Interview B1)*

But when she did receive a diagnosis, it brought her more positive feelings: she felt enabled to accept her limitations and to find constructive strategies to meet the demands of medical education.

“I started to feel better about myself… I don’t feel bad about having to print everything out now ... Whereas I used to think I was a bit picky about it, now I feel it’s OK to say … I need to touch it, I need to run my finger under it … I don’t feel bad about not being able to sit in the library for hours, which is what everyone else seems to do. I just study in short bursts, and if I’m tired, I won’t study, because I know that it won’t go in.” *(Interview B1)*

Generally speaking, those interviewed had not encountered prejudice or stigma because of their SpLD: minor instances, or fear of possible stigma, were briefly mentioned (in contrast with the third cycle, see below).Two students described an acceptance that was only partial. Student A2 described how, despite having been diagnosed at school, she found the challenges of her SpLD so normal that she wondered if she really had it.

“I even wonder myself sometimes, do I have dyslexia, am I different from anyone else, should I be getting extra time? I know I wouldn’t be here if I didn’t have extra time, but I don’t know… I can’t be like, ‘The dyslexia is annoying me’, because I don’t know anything else. So if I muddle my words, that’s just [her name] being silly.” *(Interview A2)*

This illustrates how familiarity with the SpLD can lead students to resist full acceptance of the diagnosis, and even, as in this case, to accept negative labeling (‘being silly’). This is particularly striking as this student already had both a bachelor’s degree and a doctorate.Student C10 felt unsure of the diagnosis because he had friends whose dyslexia was worse than his.

“You compare yourself to them, and it’s quite obvious that they struggle to read things and struggle to understand… it seems more obvious. So I sometimes even doubt if I am [dyslexic]…” *(Interview C10)*

For him, acceptance followed from understanding, which he felt in his case was as yet incomplete:

“I don’t even know if I fully comprehend what dyslexia is… still a learning process, I suppose.” *(Interview C10)*

Unsurprisingly, given that all students were supported by the SpLD service, there were many reports of actions taken to cope (extra time in exams, software, Dictaphones, etc.); and all had demonstrated, by continuing their studies at medical school, a commitment to pursuing their goal to become a doctor.

### After medical school

Obviously, recognition had already taken place, but the third cycle had otherwise not begun: understanding and acceptance of the impact of SpLDs on work could only be guessed at. Some students did foresee likely difficulties when working as a doctor. These included: making mistakes with drug names or drug calculations; finding acronyms difficult; writing in patient notes under pressure; encountering problems when sitting Royal College examinations.Four students did not refer to any anticipated problems in how they would do their job technically, but identified stigma as a likely problem: negative perceptions by colleagues, and a possible barrier to work progression or choice of specialty. A third group of students foresaw no problems. Some explained that this was because of aids such as Dictaphones, while others expected their own skills to have improved to a satisfactory level (e.g. learning drug names). For another, the SpLD was associated with acquiring rather than using knowledge.

“By the end of your five years, you’ve learnt all the physiology you need. So I think on the wards that will require very much sort of applying your knowledge. I’ll hopefully already know it.” *(Interview C3)*

For another, the key skills required were about interpersonal rather than written communication

“You can be a pretty good doctor as long as you just listen to the patient.” *(Interview B2)*

Another student more specifically envisaged a career in medical teaching:

“I’d probably want to get into academic, because with my dyslexia, I’d understand how students feel… students would benefit from my learning experience.” *(Interview B13)*

Expectations of the likelihood or unlikelihood of future SpLD-related difficulties were not apparently linked to membership of group A, B or C.

## Discussion

Medical training is demanding, and it may be the first time that very intelligent students find themselves struggling because of an SpLD (whether or not diagnosed previously). Some students may for the first time be comparing themselves with uniformly intelligent peers, which highlights their difficulties.Some students in this study had benefited from staff suggesting SpLD screening in response to students’ disappointing grades or reports of study difficulties. It is not clear whether staff are generally informed and helpful in this way or whether these students were lucky to encounter them. At any rate, medical schools should ensure that all staff are aware of how SpLDs may show themselves and of how university SpLD services can help students. Though there is doubtless a basic awareness of SpLDs among academic staff and a mandatory requirement that all staff receive equality and diversity training. However, each university will have its own internal system to achieve this objective and there are constraints associated with time available to academic staff to engage more fully and effectively with these issues. Detailed knowledge of students’ needs and how they can be met may be less common.Useful resources (albeit targeted at nurses and nurse managers) have been provided by the UK Royal College of Nursing^20^ which give useful information about the variety of ways in which SpLDs may show themselves and a range of support options which may be helpful.Students in this study used the Disability and Dyslexia service that exists in the university, but they reported an absence of support available to them during their clinical placements. Informants did not present this lack of support as a particular problem, though they did mention some difficulties experienced there. As healthcare attracts people with SpLDs into the professions, there will be undoubtedly qualified professionals already working in the practice settings who may be sources of support however, the Disability and Dyslexia Service does not make recommendations for clinical practice as they feel that they do not have the specialist clinical background in order to understand the environment.Stigma against students with SpLDS in the workplace has been suggested.^21^,^22^ It has also been recognised that people with SpLDs display an array of individual strengths in their profile such as: having a holistic approach, being creative thinkers, good at seeing the whole picture, having practical expertise, good spatial skills, the ability to work in 3d, resourceful, hardworking, determined, excellent verbal and or visual skills.^10^,^23^ These are not only valuable to help them to devise copying mechanisms, but they can also represent a real asset in many professions.Workplace support for those with SpLDs may be available. Dale and Aitken^5^ instance a support group for dyslexic staff at one hospital; such groups could also be open to students on placement. The General Medical Council suggests a buddy system.^24^ The Royal College of Obstetricians and Gynaecologists has advocated a mentorship model of support for disabled doctors and has given extra time in examinations to doctors with dyslexia.^9^ It would be useful to study medical students with SpLDs (as well as all medical and healthcare students) as they move into work, to identify their ongoing needs and whether these are met. Without a more objective assessment of how the students met the challenges of clinical practice, it is impossible to assess the need for such support.The fact that some did not anticipate any problems once qualified might be thought to be of some concern. Although there are reported anxieties in the literature about whether health care professionals with dyslexia can practice safely^25^-^27^ there is no robust evidence to suggest that they cannot and do not. Indeed, SpLDs are a continuum of difficulties and each individual with an SpLD has a unique profile of strengths and difficulties.^5^ Some may be more affected than others and many have developed excellent coping strategies while others are perfectly capable to do so with appropriate support. There is significant variation in the way SpLDs affect people, therefore it may be dangerous to draw general conclusions, whereas it is more appropriate to focus on each individual cognitive profile.On the basis of this study, it is plausible to suppose that students will recognise new demands when they move into post-qualification work, just as they did when they moved into medical school. Students are likely to continue ‘reframing’ their SpLD as their careers change, and therefore develop new action plans. This is, after all, only a special case of the adjustments that everyone needs to make throughout their working lives as they change roles and organisations.The data reported here show that though Gerber et al’s^16^ stages of re-framing are reflected in the students’ experience, the stages are not necessarily experienced completely, or in the designated order. Student A7, for example, had recognised and acted on SpLD before medical school, but still did not fully accept it; while several students understood and coped with learning problems without recognising that they had an SpLD.The data also illustrates the emotions associated with a process that Gerber et al^16^ describe in primarily cognitive terms. Some students may therefore need more psychological support to help them re-frame, over and above practical problem-solving help. Although the Disability and Dyslexia service provide such support, there was no evidence of this in the interviews. The help reported from the university service was practical and financial. University SpLD services might consider how to meet such needs. They could also liaise with clinical placements, in order to understand the challenges students are likely to face, and to help students devise and develop suitable strategies.The study had a number of limitations. The sample was small. It would be useful to extend the study to include more students with dyspraxia and dysgraphia as well as some with dyscalculia. Only students known to the SpLD service were included: there may be others who have not disclosed, or who are unaware that they have a SpLD^21^ or who have left the course. Identifying and including such students is desirable, but logistically challenging. The study relied on volunteers, whose reasons for participating are unknown. It is possible that those with more negative views of their SpLD chose not to put themselves in a position of having to discuss them. This may mean that students successfully using strategies of reframing were more likely to volunteer and that those most distressed are therefore not included; but this speculation needs testing. Because the focus of the interviews was the medical school experience, there are more data on the second cycle: accounts of the other two cycles involve recall or prediction and are therefore a different sort of data, arguably less robust. But the idea that successive cycles of re-framing are necessary to meet the changing conditions of study and work is an important finding.

## Conclusions

Our study shows that students with SpLDs need to recognise, understand and accept their SpLD if they are to act effectively to achieve their academic and work goals. Such a process is on-going, as demands and contexts change over time. Students in this study showed a capacity to review their SpLD and its impact as circumstances changed.What a small exploratory study cannot show is how typical this is: further research is necessary, that includes the full range of SpLDs and students who may be less willing to recognise that they have one. It would be valuable to follow students from medical school into work to see whether and how their SpLDs present new challenges, and how these are addressed.In the meantime, medical schools can try to ensure that all teaching staff are more alert to signs of undiagnosed SpLDs and see it as their responsibility to advice students of relevant services. SpLD services in universities should acquaint themselves with the challenges specific to clinical placements in order to help students meet them successfully.

### Conflict of Interest

The authors declare that they have no conflict of interest.
